# A new clinical model for predicting lymph node metastasis in T1 colorectal cancer

**DOI:** 10.1007/s00384-024-04621-y

**Published:** 2024-04-03

**Authors:** Kai Wang, Hui He, Yanyun Lin, Yanhong Zhang, Junguo Chen, Jiancong Hu, Xiaosheng He

**Affiliations:** 1 Department of Anaesthesia, The Sixth Affiliated Hospital, Sun Yat-sen University, Guangzhou, Guangdong China; 2 Department of General Surgery (Colorectal Surgery), The Sixth Affiliated Hospital, Sun Yat-sen University, Guangzhou, Guangdong China; 3 Guangdong Provincial Key Laboratory of Colorectal and Pelvic Floor Diseases, Guangdong Institute of Gastroenterology, The Sixth Affiliated Hospital, Sun Yat-sen University, Guangzhou, Guangdong China; 4 Biomedical Innovation Center, The Sixth Affiliated Hospital, Sun Yat-sen University, Guangzhou, Guangdong China; 5 Department of Thoracic Surgery, Thoracic Cancer Center, The Sixth Affiliated Hospital, Sun Yat-sen University, Guangzhou, Guangdong China

**Keywords:** T1 colorectal cancer (CRC), Lymph node metastasis (LNM), Prediction, Sex, Depth of submucosal invasion (DSI)

## Abstract

**Purpose:**

Lymph node metastasis (LNM) is a crucial factor that determines the prognosis of T1 colorectal cancer (CRC) patients. We aimed to develop a practical prediction model for LNM in T1 CRC.

**Methods:**

We conducted a retrospective analysis of data from 825 patients with T1 CRC who underwent radical resection at a single center in China. All enrolled patients were randomly divided into a training set and a validation set at a ratio of 7:3 using R software. Risk factors for LNM were identified through multivariate logistic regression analyses. Subsequently, a prediction model was developed using the selected variables.

**Results:**

The lymph node metastasis (LNM) rate was 10.1% in the training cohort and 9.3% in the validation cohort. In the training set, risk factors for LNM in T1 CRC were identified, including depressed endoscopic gross appearance, sex, submucosal invasion combined with tumor grade (DSI-TG), lymphovascular invasion (LVI), and tumor budding. LVI emerged as the most potent predictor for LNM. The prediction model based on these factors exhibited good discrimination ability in the validation sets (AUC: 79.3%). Compared to current guidelines, the model could potentially reduce over-surgery by 48.9%. Interestingly, we observed that sex had a differential impact on LNM between early-onset and late-onset CRC patients.

**Conclusions:**

We developed a clinical prediction model for LNM in T1 CRC using five factors that are easily accessible in clinical practice. The model has better predictive performance and practicality than the current guidelines and can assist clinicians in making treatment decisions for T1 CRC patients.

**Supplementary Information:**

The online version contains supplementary material available at 10.1007/s00384-024-04621-y.

## Introduction

Colorectal cancer (CRC) ranks as the third most prevalent malignant tumor globally and stands as the second leading cause of cancer-related deaths [1]. Its incidence and mortality rates persistently rise, substantially adding to the overall burden of cancer worldwide [2–4]. There is a growing recognition of the importance of screening colonoscopy and other preventive measures for CRC in an expanding array of countries [5, 6].

Advancements in endoscopic techniques have resulted in a heightened detection rate of T1 CRC and an increased number of endoscopic resections [7]. Endoscopic resection is considered a curable approach if there is no evidence of lymph node metastasis (LNM) [8]. However, patients with a risk of LNM must undergo radical surgery after endoscopic resection to reduce the risk of cancer recurrence [9]. Hence, accurate assessment of the likelihood of LNM in T1 CRC patients is pivotal for guiding treatment decisions.

In the guidelines from the United States, Europe, Korea, and Japan, certain factors are designated as high-risk for lymph node metastasis (LNM) in T1 colorectal cancer (CRC), warranting surgical resection. These factors include a depth of submucosal invasion (DSI) of ≥ 1000 μm, lymphovascular invasion (LVI), tumor grade (TG, G3-poorly differentiated adenocarcinoma, signet-ring cell carcinoma, or mucinous carcinoma) and tumor budding (TB, BD2/3) [10–17]. However, it is noteworthy that only about 10% of patients identified as high-risk based on these guidelines actually exhibit LNM, with the vast majority (> 90%) eventually showing negative lymph nodes upon histological examination of the surgical specimen [18–23]. This discrepancy underscores the limitations of current guidelines, which fail to consider additional risk factors. The binary nature of these guidelines leads to significant overtreatment, placing a strain on clinical healthcare resources. Given these challenges, there is an urgent need for a clinical prognostic model that integrates more dependable predictors to customize optimal treatment strategies for T1 CRC patients.

This study analyzed the risk factors for LNM in 825 patients with T1 CRC from a single center in China. Subsequently, a clinical prediction model was established, requiring only additional data on endoscopic gross appearance and the objective factor of sex based on the guidelines. The model has demonstrated good predictive performance and practicality, and it is expected to be clinically utilized to aid in treatment selection for T1 CRC patients.

## Materials and methods

### Study design and population

**Fig. 1:**
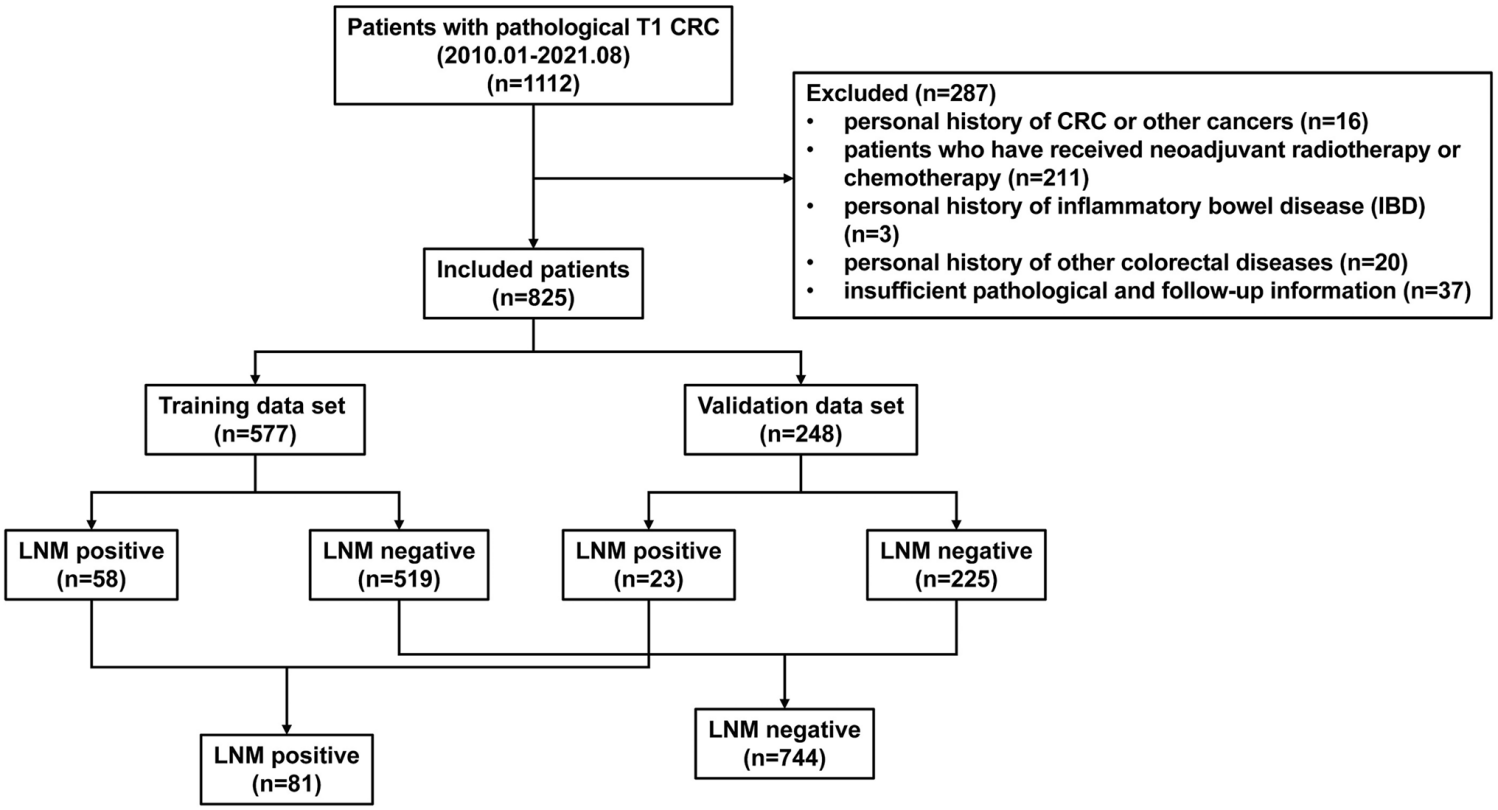
Consort diagram of collected T1 colorectal cancers (CRCs). *LN* Lymph node, *LNM* lymph node metastasis

Patient data for this study were obtained from the prospectively maintained institutional database program of colorectal disease at the Sixth Affiliated Hospital, Sun Yat-sen University (Guangzhou, China). All patients over 18 years of age with primary T1 CRC who had received radical tumor resection between January 2010 and August 2021 were enrolled. Inclusion criteria were (1) histopathologically confirmed colorectal adenocarcinoma; (2) pathologically diagnosed T1 without distal metastasis; (3) radical resection was performed with or without previous endoscopic resection. Exclusion criteria were as follows: (1) personal history of CRC or other cancers; (2) patients who have received neoadjuvant radiotherapy or chemotherapy; (3) personal history of inflammatory bowel disease (IBD); (4) personal history of other colorectal diseases, such as familial adenomatous polyposis (FAP) or Peutz-Jeghers syndrome (PJS); (5) insufficient pathological and follow-up information. To enhance the robustness of the analysis, all enrolled patients were randomly divided into a training set and a validation set at a ratio of 7:3 using R software, as illustrated in Fig. 1. Furthermore, patients were categorized into lymph node metastasis negative (LNM-negative) and lymph node metastasis positive (LNM-positive) groups based on the presence of lymph node metastasis for subsequent analysis. This study protocol received approval from the Ethics Review Committee of the Sixth Affiliated Hospital, Sun Yat-sen University (2022ZSLYEC-120).

### Data collection

A comprehensive collection of demographic, clinical, surgical, and post-operative data was undertaken by proficient assistants from the institutional database. Additionally, the accuracy of pathological information was ensured through confirmation by a minimum of two experienced pathologists. In this study, we focused on a selection of risk factors demonstrated to be potentially linked to lymph node metastasis (LNM) in T1 colorectal cancer (CRC) patients. The chosen factors included age, sex, endoscopic gross appearance, tumor grade (TG), depth of submucosal invasion (DSI), tumor budding (TB), lymphovascular invasion (LVI), tumor location, tumor size, as well as the combination of DSI with TG (DSI-TG).

### Study definitions

All pathology reports from enrolled patients were reviewed for the presence of LNM. In this study, patients with an age of CRC onset younger than 50 were defined as early-onset CRC (EOCRC) patients. According to the Paris classification of endoscopic findings of superficial colorectal neoplasms, the tumors were morphologically classified into depressed and undepressed [24]. A lesion of IIc or III classification in the Paris classification was defined as a depressed lesion. Tumor grade (TG) was classified according to the histologic type. In brief, G1 was assigned for tumors diagnosed as papillary adenocarcinoma and well-differentiated tubular adenocarcinoma, G2 to moderately differentiated adenocarcinoma, and G3 to poorly differentiated adenocarcinoma, mucinous adenocarcinoma, or signet ring cell carcinoma. Regarding the measurement of depth of submucosal invasion (DSI), the depth was measured from the lower margin of the mucosal muscle to the deepest invasion, provided the location of the mucosal muscle could still be identified or estimated. If the mucosal muscle was completely absent, the depth was measured from the surface to the deepest invasion edge. DSI as an independent predictor of LMN is controversial. Based on the results of previous studies, we explored the prediction effectiveness using the composite indicator of DSI with TG. Tumor budding (TB) was defined as a cancer cell nest consisting of 1 to < 5 cells that infiltrated the interstitium at the invasive margin of cancer. TB was graded based on the number of buds as BD1 (< 5), BD2 (5–9), or BD3 (≥ 10), as previous research had reported.

### Statistical analyses

Continuous variables were compared using either Student’s *t*-test or Wilcoxon rank-sum test, while the Chi-squared test was used for discrete variables to compare the distribution characteristics. We performed a univariate logistic regression model to estimate the association between risk factors and LNM. All variables with a *P* value < 0.05 were further fitted into a multivariate model in the “enter” way. Additionally, ROC curve analysis was applied to test the prediction ability of the model in the training set and validation set. All statistical analyses were performed using SPSS software (Version 22.0) and R software (Version 4.0.0). All statistical tests were two-sided, and a *P* value < 0.05 was considered statistically significant.

## Results

### Characteristics of the training and validation cohorts

The clinical characteristics of both the training and validation sets are summarized in Table 1. The training set comprised 577 patients, while the validation set included 248 patients, making a total of 825 enrolled individuals. The rate of lymph node metastasis (LNM) was 10.1% in the training cohort and 9.3% in the validation cohort, with no statistically significant difference observed (*P* = 0.731). In the training set, the mean age was 58.5 years, with a male-to-female ratio of 55.5:44.5. Among the 577 lesions, 85 (14.7%) were characterized as depressed type, and 42 (7.3%) exhibited submucosal invasion of less than 1000 μm. BD1 was observed in 514 (89.1%) tumors, and 545 (94.5%) lesions were without lymphovascular invasion (LVI). The clinicopathological characteristics were comparable between the training and validation sets.

**Table 1:** Clinicopathologic characteristics of the training and validation data sets

Parameter	Category	Training data set (*n* = 577)	Validation data set (*n* = 248)	*P* value*
Age, y	Mean	58.5	58.1	0.116
	Range	27–88	27–86	
Sex, *n* (%)	Male	320(55.5)	143(57.7)	0.559
	Female	257(44.5)	105(42.3)	
Endoscopic gross appearance, n (%)	Depressed	85(14.7)	46(18.6)	0.169
	Undepressed	492(85.3)	202(81.4)	
Tumor grade, *n* (%)	G1	161(27.9)	80(32.2)	0.426
	G2	373(64.6)	149(60.1)	
	G3	43(7.5)	19(7.7)	
Depth of submucosal invasion, *n* (%)	< 1000 μm	42(7.3)	18(7.3)	0.967
	1000–1999 μm	83(14.4)	34(13.7)	
	≥ 2000 μm	452(78.3)	196(79.0)	
Tumor budding, *n* (%)	BD1	514(89.1)	222(89.5)	0.854
	BD2/BD3	63(10.9)	26(10.5)	
Lymphovascular invasion, *n* (%)	Negative	545(94.5)	232(93.6)	0.610
	Positive	32(5.5)	16(6.4)	
Tumor location, *n* (%)	R	297(51.5)	139(56.0)	0.330
	D/S	198(34.3)	82(33.1)	
	T/A/C	82(14.2)	27(10.9)	
Tumor size, mm	Mean	22.8	21.5	0.459
	Range	4.0–120.0	5.0–77.0	
Lymph node metastasis, *n* (%)	Negative group	519(89.9)	225(90.7)	0.731
	Positive group	58(10.1)	23(9.3)	

*R* rectum, *D* descending colon, *S* sigmoid colon, *T* transverse colon, *A* ascending colon, *C* cecum

*Student *t* test (age and tumor size) or *χ*^2^ test (other categorical variables)

### Risk factors for LNM and development of a predictive model

The results of the univariate analysis for the training set are presented in Table 2. Consistent with established guidelines, LVI, TB (BD2/BD3), and TG (G3) were identified as risk factors for LNM. Interestingly, DSI(DSI ≥ 1000 μm) was not identified as a standalone risk factor, but when combined with G2/G3 to analysis, it demonstrated significance. A logistic regression prediction model was constructed using the six variables demonstrating significant associations with LNM in Table 2. TG was excluded from the regression model due to multicollinearity (i.e., less specificity and high correlation with DSI-TG), which could potentially diminish the statistical significance of the model. Consequently, the model comprised five independent factors (depressed endoscopic gross appearance, sex, DSI-TG, LVI, TB) as LNM predictors. LVI emerged as the most potent predictor for LNM, increasing the incidence of metastasis tenfold (OR, 10.369; 95%CI, 4.60–23.28), depressed endoscopic g ross appearance (OR,2.820; 95%CI,1.41–5.48), sex (OR, 0.564; 95%CI,0.31–1.00), DSI-TG (OR, 1.960; 95%CI,0.99–4.20), TB (OR, 1.387; 95%CI, 0.61–2.94). Furthermore, a guideline-combination model was developed, incorporating the four guideline factors: DSI, LVI, TG, and TB.

**Table 2:** Univariate for selected risk factors and logistic regression model to predict for LNM in T1 colorectal cancer (training data set)

			Univariate			Multivariate	
Parameter	Category	No.	LNM-positive group(*n* = 58)	LNM-negative group(*n* = 519)	*P* value	Odds ratio(95% CI)	*P* value
Age	< 50	111	13(11.7%)	98(88.3%)	0.518	-	-
	≥ 50	466	45(9.7%)	421(90.3%)		-	-
Sex	Female	257	33(12.8%)	224(87.2%)	0.048	Reference	
	Male	320	25(7.8%)	295(92.2%)		0.564(0.31–1.00)	0.054
Endoscopic gross appearance	Undepressed	492	42(8.5%)	450(91.5%)	0.005	Reference	
	Depressed	85	16(18.8%)	69(81.2%)		2.820(1.41–5.48)	0.002
Tumor grade	G1	161	10(6.2%)	151(93.8%)		-	-
	G2	373	41(11.0%)	332(89.0%)	0.089	-	-
	G3	43	7(16.3%)	36(83.7%)	0.041	-	-
Depth of submucosal invasion	< 1000 μm	42	6(14.3%)	36(85.7)		-	-
	1000–1999 μm	83	8(9.6%)	75(90.4%)	0.439	-	-
	≥ 2000 μm	452	44(9.7%)	408(90.3%)	0.353	-	-
Tumor budding	BD1	514	47(9.1%)	467(90.9%)	0.042	Reference	
	BD2/BD3	63	11(17.5%)	52(82.5%)		1.387(0.61–2.94)	0.413
Lymphovascular invasion	Negative	545	44(8.1%)	501(91.9%)	< 0.001	Reference	
	Positive	32	14(43.8%)	18(56.2%)		10.369(4.60–23.28)	< 0.001
Tumor location	R	297	35(11.8%)	262(88.2%)		-	-
	D/S	198	16(8.1%)	182(91.9%)	0.187	-	-
	T/A/C	82	7(8.5%)	75(91.5%)	0.409	-	-
Tumor size	1–14 mm	88	6(6.8%)	82(93.2%)	0.277	-	-
	≥ 15 mm	489	52(10.6%)	437(89.4%)		-	-
DSI-TG	Others	184	11(6.0%)	173(94%)	0.029	Reference	
	G2/G3 & ≥ 1000 μm	393	47(12.0%)	346(88.0%)		1.960(0.99–4.20)	0.065

*LNM* lymph node metastasis, *CI* confidence interval, *R* rectum, *D* descending colon, *S* sigmoid colon, *T* transverse colon, *A* ascending colon, *C* cecum, *DSI* depth of submucosal invasion, *TG* tumor grade, *DSI-TG* depth of submucosal invasion combined with tumor grade

Additionally, sex and the endoscopic gross appearance of depressed type were recognized as additional risk factors for LNM. Female gender is a risk factor for lymph node metastasis in T1 colorectal cancer. However, the role of gender factors is not consistent in early versus late onset populations. In T1 late-onset colorectal cancer (LOCRC), the rate of LNP in female patients (12.8%) was higher than that in male patients (7.2%; *P* = 0.049). Notably, sex did not exert an impact on LNM in T1 EOCRC patients (*P* = 0.640), as detailed in Table 3. Supplementary Table 1 provides the clinical-pathological characteristics of EOCRC and LOCRC.

**Table 3:** Univariable analysis about sex for predicting LNM in EOCRC and LOCRC

Parameter	Category	No.	LNM-positive	LNM-negative	*P* value
Early-onset	Male	58	6(10.3%)	52(89.7%)	0.640
	Female	53	7(13.2%)	46(86.8%)	
Late-onset	Male	262	19(7.2%)	243(92.8%)	0.049
	Female	204	26(12.8%)	178(87.3%)	

*LNM* lymph node metastasis, *EOCRC* early-onset colorectal cancer, *LOCRC* late-onset colorectal cancer

The endoscopic gross appearance of depressed type was another additional risk factors for LNM. The tissue with the depressed appearance was found to have a more superficial DSI as outlined in Supplementary Table 2.

### Overall performance of the prediction model

**Fig. 2:**
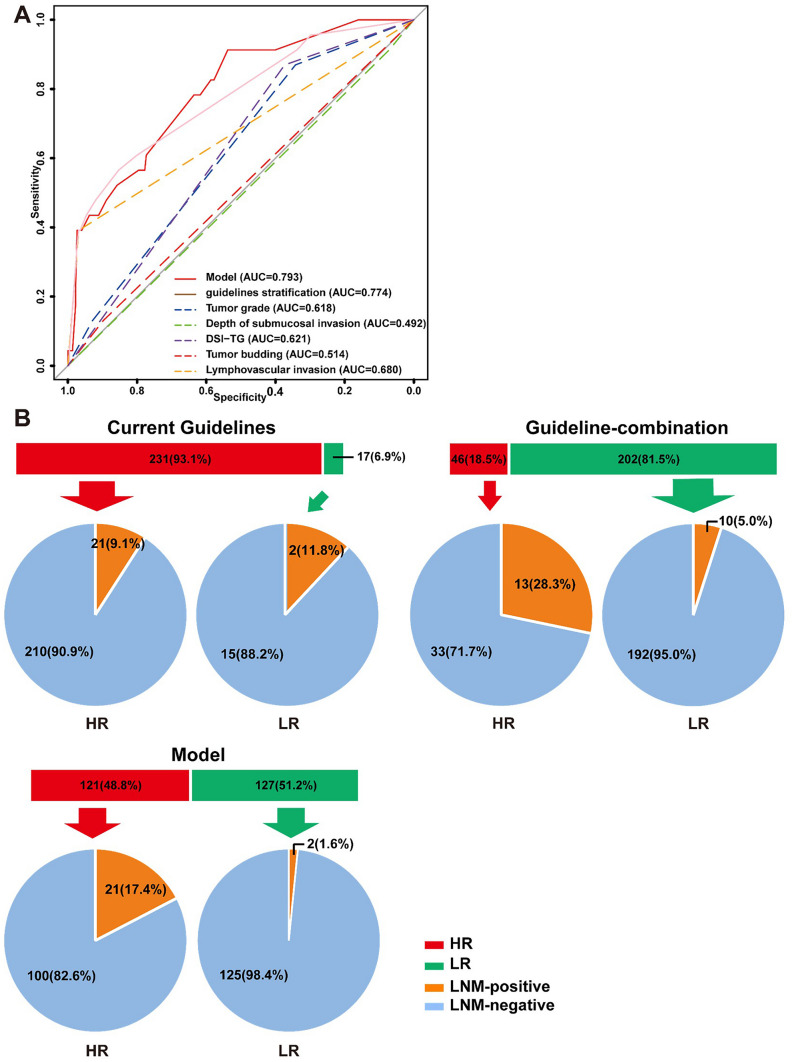
Performance evaluation of the prediction model in predicting lymph node metastasis from validation data set. **A** A receiver operating characteristic curve analysis to compare the performance of the risk factors of current guidelines, guideline-combination model and the prediction model in a validation cohort. **B** Comparison of overtreatment frequency among current guidelines, guideline-combination model and our prediction model. *HR* high risk, *LR* low risk, *LNM* lymph node metastasis

To validate its reliability, the Hosmer-Lemeshow statistic for the model was 2.379 (*P* = 0.795). When predicting the risk of LNM in the validation set using risk factors from current guidelines, Fig. 2 illustrates that 231 out of 248 lesions were categorized as high risk, while 17 were deemed low risk. Among the high-risk group, 21 lesions (9.1%) exhibited LNM, whereas in the low-risk group, 2 lesions (11.8%) showed LNM. Remarkably, the guideline risk factors did not accurately differentiate lesions with LNM. The guideline-combination model, aiming to avoid an all-or-nothing decision, assigned 202 patients to the low-risk group. However, 43.5% (10 out of 23) of positive patients in the low-risk group were misdiagnosed. Utilizing the predictive model developed in this study, 121 patients in the validation set were classified as high risk, among whom 21 (17.4%) had LNM. In contrast, only 2 (1.6%) of the 127 patients in the low-risk group exhibited LNM. Importantly, a mere 8.7% (2 out of 23) of patients who tested positive were categorized as low-risk group according to both this prediction model and the current guidelines. These findings suggest that our model offers superior risk stratification for T1 CRC patients with LNM.

## Discussion

This retrospective study analyzed the risk factors for LNM in 825 T1 CRC patients from a single center in China. Based on the clinical-pathological data, a clinical prediction model was established. The univariate analysis revealed that female, depressed endoscopic gross appearance, LVI, TB, TG and DSI-TG were risk factors for LNM in T1 CRC. Subsequently, a clinical prediction model was developed, which incorporated endoscopic gross appearance and sex, in addition to the factors outlined in the guidelines. The prediction model adjusted the application of risk factors according to the guidelines and included two easily accessible additional factors. Compared to the current guidelines, the model could potentially reduce over-surgery by 48.9%. Therefore, this clinical prediction model is both practical and demonstrates superior predictive performance (AUC = 0.793).

In the validation data set, our model exhibited superior capabilities in stratifying the risk of LNM in T1 CRC patients. According to prevailing guidelines, 231 patients (93.1%) in our study cohort were recommended for curative surgical treatment. However, only 21 patients (9.5%) manifested LNM, suggesting that 210 patients (90.5%) may have undergone unnecessary treatment. Across various cohort studies, an estimated 80–90% of patients are reported to experience overtreatment based on guideline criteria [22, 25–27]. Initially, we formulated a predictive model relying on guideline-based factors (guideline-combination model), avoiding the all-or-nothing decision-making approach that categorized 202 patients (81.5%) into the low-risk group. However, this model, with lower sensitivity, exhibited a notable false-negative rate of 43.5% for positive patients. Subsequently, following a detailed analysis of guideline and non-guideline risk factors, our clinical prediction model, integrating the DSI-TG risk factor and two easily assessable preoperational elements—sex and endoscopic gross appearance—displayed enhanced prediction accurate rate (98.4%) while concurrently mitigating overtreatment in almost half of the patients. The ease of obtaining additional factors further amplifies the clinical applicability of our clinical prediction model.

A recent meta-analysis has highlighted that DSI is not an independent risk factor for LNM in T1 CRC but holds predictive significance when analyzed in conjunction with other risk factors [28]. Consistent with this finding, our study corroborates that DSI exhibits enhanced predictive efficacy when combined with tumor grade (DSI-TG, AUC = 0.621; DSI, AUC = 0.492). While previous studies have endeavored to enhance the predictive efficacy of DSI by modifying its assessment criteria, current evaluation models for determining the depth of submucosal invasion in early-stage colorectal cancer primarily include the Haggitt classification and Kikuchi-SM system. These models are recommended for assessing the risk of pedunculated and sessile polyps, respectively [17].

Kikuchi et al. introduced a novel model that categorizes the depth of tumor submucosal invasion based on its approximate distance from the muscularis mucosae [29]. According to this model, superficial invasion (within 200–300 μm of the muscularis mucosae) is designated as SM1, while deep invasion (proximal to the muscularis propria) is classified as SM3. Depths of infiltration falling between SM1 and SM3 are denoted as SM2. This model was employed to reevaluate the depth of submucosal invasion in patients with early-stage colorectal cancer. However, it’s noteworthy that the precise definition of SM1 to SM3 within the SM system can vary among different studies, potentially introducing subjective variability and impacting the clinical applicability of the model. Our study refrained from altering the assessment criteria for this pathological feature, minimizing the impact of subjective variability on the model’s practicality.

Endoscopic screening plays a pivotal role in diagnosing early-stage CRC [5, 30]. Recent research has begun to explore the significance of endoscopic gross appearance in predicting LNM in T1 CRC patients. Our study identified the gross appearance of depressed type as a risk factor for LNM in T1 CRC. While guidelines mainly rely on postoperative pathological characteristics, evaluating endoscopic gross appearance can complement preoperative assessments. The classification of endoscopic gross appearance into the depressed type is based on the simplified Paris classification, facilitating its straightforward clinical acquisition and ensuring the practicality of our clinical prediction model. Additionally, the depressed type of endoscopic gross appearance is associated with a higher risk of LNM and a shallower average DSI. DSI is one of the criteria for surgical resection in the guidelines. This finding underscores the importance of reassessing the DSI criterion concerning different endoscopic gross appearances.

Our study revealed that protective factors in female patients with T1 CRC are often overlooked. Despite numerous cohort studies examining objective risk factors, the impact of gender on LNM in T1 CRC remains contentious, with inconsistent findings reported [19, 21, 31]. A pertinent meta-analysis suggests a higher likelihood of LNM among female patients with T1 CRC [32]. However, several studies underscore the protective influence of estrogen in CRC, challenging the notion that females are inherently more susceptible to LNM in T1 CRC [33–35]. In our study, the results of univariate analysis confirmed the gender differences across age groups, with sex having no effect on LNM in T1 EOCRC. Conversely, in T1 LOCRC, female emerged as a risk factor for LNM (P = 0.049). It appears that there exists a notable contrast in estrogen levels between female T1 EOCRC and female T1 LOCRC patients. This divergence may account for the distinct influence of sex on LNM in the two patient groups. A population-based data study conducted in the United States on the risk factors for LNM in young T1 CRC patients also supports this conclusion [36]. They found that the overall LNM rate in T1 CRC was approximately 22% in young patients (less than 45 years old), with a slightly higher incidence in females. Tumor size and tumor grade were significant predictors of LNM in T1 CRC cancer patients. However, gender was not found to be an independent predictor of LNM in young patients. The compositional bias in these two patient groups might contribute to the differing research conclusions on the objective risk factor of sex. As the incidence of EOCRC continues to rise and the incidence among the elderly declines, it has become a new global trend in CRC epidemiology [30, 37]. Therefore, in subsequent more data of multi-center cohort studies developing risk scoring systems, it is imperative to conduct separate investigations for EOCRC and LOCRC, representing a key focus for our future work.

The current study has some limitations. Firstly, it lacks an external validation cohort for prospective assessment, relying solely on data from a single center. Further validation from multi-center sources would enhance the robustness and generalizability of the findings. Secondly, there is minimal missing data. Thirdly, the restricted sample size hampers the development of predictive models for T1 EOCRC and T1 LOCRC separately. However, our clinical prediction model exhibited significant predictive power in both groups (Supplementary Fig. 1). This is attributed to the considerably larger number of patients with LOCRC than EOCRC, while the effect of gender is less pronounced. Thus, while the clinical prediction model in this study exhibits practical utility, its efficacy necessitates further refinement and validation. Currently, it primarily serves as a tool to aid clinicians in formulating treatment strategies for T1 CRC patients.

We developed a clinical prediction model for lymph node metastasis in T1 colorectal cancer patients, based on five factors: sex, endoscopic gross appearance, depth of submucosal invasion combined with tumor grade, lymphovascular invasion, and tumor budding. The model improved the accuracy and practicality of risk stratification, compared to the current guidelines, and reduces overtreatment by almost half. We also identified the protective role of female sex in T1 colorectal cancer. We suggested that early-onset and late-onset colorectal cancer patients should be analyzed separately. Further validation from multi-center prospective studies is warranted.

## Supplementary Information

Below is the link to the electronic supplementary material.

Supplementary file1 (TIF 19796 KB)

Supplementary file2 (XLSX 11 KB)

Supplementary file3 (XLSX 10 KB)

## Data Availability

The data supporting the findings of this study are available from the corresponding author upon reasonable request.

## References

[1] GBD (2019) Diseases and Injuries Collaborators (2020) Global burden of 369 diseases and injuries in 204 countries and territories, 1990–2019: a systematic analysis for the Global Burden of Disease Study 2019. Lancet 396:1204–1222. 10.1016/S0140-6736(20)30925-9 10.1016/S0140-6736(20)30925-9 PMC7567026 33069326

[2] Morgan E, Arnold M, Gini A et al (2023) Global burden of colorectal cancer in 2020 and 2040: incidence and mortality estimates from GLOBOCAN. Gut 72:338–344. 10.1136/gutjnl-2022-327736 36604116 10.1136/gutjnl-2022-327736

[3] Keum N, Giovannucci E (2019) Global burden of colorectal cancer: emerging trends, risk factors and prevention strategies. Nat Rev Gastroenterol Hepatol 16:713–732. 10.1038/s41575-019-0189-8 31455888 10.1038/s41575-019-0189-8

[4] Qu R, Ma Y, Zhang Z, Fu W (2022) Increasing burden of colorectal cancer in China. Lancet Gastroenterol Hepatol 7:700. 10.1016/S2468-1253(22)00156-X 35809603 10.1016/S2468-1253(22)00156-X

[5] Ladabaum U, Dominitz JA, Kahi C, Schoen RE (2020) Strategies for colorectal cancer screening. Gastroenterology 158:418–432. 10.1053/j.gastro.2019.06.043 31394083 10.1053/j.gastro.2019.06.043

[6] Bretthauer M, Løberg M, Wieszczy P et al (2022) Effect of colonoscopy screening on risks of colorectal cancer and related death. N Engl J Med 387:1547–1556. 10.1056/NEJMoa2208375 36214590 10.1056/NEJMoa2208375

[7] Bretthauer M, Kaminski MF, Løberg M et al (2016) Population-based colonoscopy screening for colorectal cancer: a randomized clinical trial. JAMA Intern Med 176:894–902. 10.1001/jamainternmed.2016.0960 27214731 10.1001/jamainternmed.2016.0960 PMC5333856

[8] Tamaru Y, Oka S, Tanaka S et al (2017) Long-term outcomes after treatment for T1 colorectal carcinoma: a multicenter retrospective cohort study of Hiroshima GI Endoscopy Research Group. J Gastroenterol 52:1169–1179. 10.1007/s00535-017-1318-1 28194526 10.1007/s00535-017-1318-1

[9] Yamashita K, Oka S, Tanaka S et al (2019) Preceding endoscopic submucosal dissection for T1 colorectal carcinoma does not affect the prognosis of patients who underwent additional surgery: a large multicenter propensity score-matched analysis. J Gastroenterol 54:897–906. 10.1007/s00535-019-01590-w 31104172 10.1007/s00535-019-01590-w

[10] Park CH, Yang D-H, Kim JW et al (2020) Clinical practice guideline for endoscopic resection of early gastrointestinal cancer. Clin Endosc 53:142–166. 10.5946/ce.2020.032 32252507 10.5946/ce.2020.032 PMC7137564

[11] Benson AB, Venook AP, Cederquist L et al (2017) Colon cancer, Version 1.2017, NCCN Clinical Practice Guidelines in Oncology. J Natl Compr Canc Netw 15:370–398. 10.6004/jnccn.2017.0036 28275037 10.6004/jnccn.2017.0036

[12] Labianca R, Nordlinger B, Beretta GD et al (2013) Early colon cancer: ESMO Clinical Practice Guidelines for diagnosis, treatment and follow-up. Ann Oncol 24(Suppl 6):vi64-72. 10.1093/annonc/mdt354 24078664 10.1093/annonc/mdt354

[13] Kaltenbach T, Anderson JC, Burke CA et al (2020) Endoscopic removal of colorectal lesions-recommendations by the US Multi-Society Task Force on Colorectal Cancer. Gastroenterology 158:1095–1129. 10.1053/j.gastro.2019.12.018 32122632 10.1053/j.gastro.2019.12.018

[14] Pimentel-Nunes P, Libânio D, Bastiaansen BAJ et al (2022) Endoscopic submucosal dissection for superficial gastrointestinal lesions: European Society of Gastrointestinal Endoscopy (ESGE) Guideline - Update 2022. Endoscopy 54:591–622. 10.1055/a-1811-7025 35523224 10.1055/a-1811-7025

[15] Hashiguchi Y, Muro K, Saito Y et al (2020) Japanese Society for Cancer of the Colon and Rectum (JSCCR) guidelines 2019 for the treatment of colorectal cancer. Int J Clin Oncol 25:1–42. 10.1007/s10147-019-01485-z 31203527 10.1007/s10147-019-01485-z PMC6946738

[16] Benson AB, Venook AP, Al-Hawary MM et al (2018) Rectal cancer, Version 2.2018, NCCN Clinical Practice Guidelines in Oncology. J Natl Compr Canc Netw 16:874–901. 10.6004/jnccn.2018.0061 30006429 10.6004/jnccn.2018.0061 PMC10203817

[17] Glynne-Jones R, Wyrwicz L, Tiret E et al (2017) Rectal cancer: ESMO Clinical Practice Guidelines for diagnosis, treatment and follow-up. Ann Oncol 28:iv22–iv40. 10.1093/annonc/mdx224 28881920 10.1093/annonc/mdx224

[18] Nishida T, Egashira Y, Akutagawa H et al (2014) Predictors of lymph node metastasis in T1 colorectal carcinoma: an immunophenotypic analysis of 265 patients. Dis Colon Rectum 57:905–915. 10.1097/DCR.0000000000000168 25003285 10.1097/DCR.0000000000000168

[19] Yasue C, Chino A, Takamatsu M et al (2019) Pathological risk factors and predictive endoscopic factors for lymph node metastasis of T1 colorectal cancer: a single-center study of 846 lesions. J Gastroenterol 54:708–717. 10.1007/s00535-019-01564-y 30810812 10.1007/s00535-019-01564-y

[20] Oh JR, Park B, Lee S et al (2019) Nomogram development and external validation for predicting the risk of lymph node metastasis in T1 colorectal cancer. Cancer Res Treat 51:1275–1284. 10.4143/crt.2018.569 30653743 10.4143/crt.2018.569 PMC6790837

[21] Kajiwara Y, Oka S, Tanaka S et al (2023) Nomogram as a novel predictive tool for lymph node metastasis in T1 colorectal cancer treated with endoscopic resection: a nationwide, multicenter study. Gastrointest Endosc 97:1119-1128.e5. 10.1016/j.gie.2023.01.022 36669574 10.1016/j.gie.2023.01.022

[22] Miyazaki K, Wada Y, Okuno K et al (2023) An exosome-based liquid biopsy signature for pre-operative identification of lymph node metastasis in patients with pathological high-risk T1 colorectal cancer. Mol Cancer 22:2. 10.1186/s12943-022-01685-8 36609320 10.1186/s12943-022-01685-8 PMC9817247

[23] Kang J, Choi YJ, Kim I-K et al (2021) LASSO-based machine learning algorithm for prediction of lymph node metastasis in T1 colorectal cancer. Cancer Res Treat 53:773–783. 10.4143/crt.2020.974 33421980 10.4143/crt.2020.974 PMC8291173

[24] Kudo S-E, Kouyama Y, Ogawa Y et al (2020) Depressed colorectal cancer: a new paradigm in early colorectal cancer. Clin Transl Gastroenterol 11:e00269. 10.14309/ctg.0000000000000269 33512809 10.14309/ctg.0000000000000269 PMC7732270

[25] Piao ZH, Ge R, Lu L (2023) An artificial intelligence prediction model outperforms conventional guidelines in predicting lymph node metastasis of T1 colorectal cancer. Front Oncol 13:1229998. 10.3389/fonc.2023.1229998 37941556 10.3389/fonc.2023.1229998 PMC10628635

[26] Song JH, Hong Y, Kim ER et al (2022) Utility of artificial intelligence with deep learning of hematoxylin and eosin-stained whole slide images to predict lymph node metastasis in T1 colorectal cancer using endoscopically resected specimens; prediction of lymph node metastasis in T1 colorectal cancer. J Gastroenterol 57:654–666. 10.1007/s00535-022-01894-4 35802259 10.1007/s00535-022-01894-4

[27] Takashina Y, Kudo S-E, Kouyama Y et al (2023) Whole slide image-based prediction of lymph node metastasis in T1 colorectal cancer using unsupervised artificial intelligence. Dig Endosc 35:902–908. 10.1111/den.14547 36905308 10.1111/den.14547

[28] Zwager LW, Bastiaansen BAJ, Montazeri NSM et al (2022) Deep submucosal invasion is not an independent risk factor for lymph node metastasis in T1 colorectal cancer: a meta-analysis. Gastroenterology 163:174–189. 10.1053/j.gastro.2022.04.010 35436498 10.1053/j.gastro.2022.04.010

[29] Kikuchi R, Takano M, Takagi K et al (1995) Management of early invasive colorectal cancer. Risk of recurrence and clinical guidelines. Dis Colon Rectum 38:1286–1295. 10.1007/BF02049154 7497841 10.1007/BF02049154

[30] Patel SG, Karlitz JJ, Yen T et al (2022) The rising tide of early-onset colorectal cancer: a comprehensive review of epidemiology, clinical features, biology, risk factors, prevention, and early detection. Lancet Gastroenterol Hepatol 7:262–274. 10.1016/S2468-1253(21)00426-X 35090605 10.1016/S2468-1253(21)00426-X

[31] Miyachi H, Kudo S, Mochizuki K et al (2020) Tumor location and patient sex are novel risk factors of lymph node metastasis in T1 colorectal cancer. J Gastroenterol Hepatol 35:2292. 10.1111/jgh.15242 32875604 10.1111/jgh.15242

[32] Ichimasa K, Kudo S-E, Miyachi H et al (2017) Patient gender as a factor associated with lymph node metastasis in T1 colorectal cancer: a systematic review and meta-analysis. Mol Clin Oncol 6:517–524. 10.3892/mco.2017.1172 28413659 10.3892/mco.2017.1172 PMC5374909

[33] Simon MS, Chlebowski RT, Wactawski-Wende J et al (2012) Estrogen plus progestin and colorectal cancer incidence and mortality. J Clin Oncol 30:3983–3990. 10.1200/JCO.2012.42.7732 23008295 10.1200/JCO.2012.42.7732 PMC3488271

[34] Williams C, DiLeo A, Niv Y, Gustafsson J-Å (2016) Estrogen receptor beta as target for colorectal cancer prevention. Cancer Lett 372:48–56. 10.1016/j.canlet.2015.12.009 26708506 10.1016/j.canlet.2015.12.009 PMC4744541

[35] Paganini-Hill A (1999) Estrogen replacement therapy and colorectal cancer risk in elderly women. Dis Colon Rectum 42:1300–1305. 10.1007/BF02234219 10528768 10.1007/BF02234219

[36] Ramai D, Singh J, Facciorusso A et al (2021) Predictors of lymph node metastasis in T1 colorectal cancer in young patients: results from a National Cancer Registry. J Clin Med 10:5511. 10.3390/jcm10235511 34884212 10.3390/jcm10235511 PMC8658610

[37] Ugai T, Sasamoto N, Lee H-Y et al (2022) Is early-onset cancer an emerging global epidemic? Current evidence and future implications. Nat Rev Clin Oncol 19:656–673. 10.1038/s41571-022-00672-8 36068272 10.1038/s41571-022-00672-8 PMC9509459

