# Supplementary figures and images for: A new clinical model for predicting lymph node metastasis in T1 colorectal cancer
Source: Int J Colorectal Dis. 2024 Apr 3;39(1):46. doi: 10.1007/s00384-024-04621-y (PMC10987358; doi:10.1007/s00384-024-04621-y)

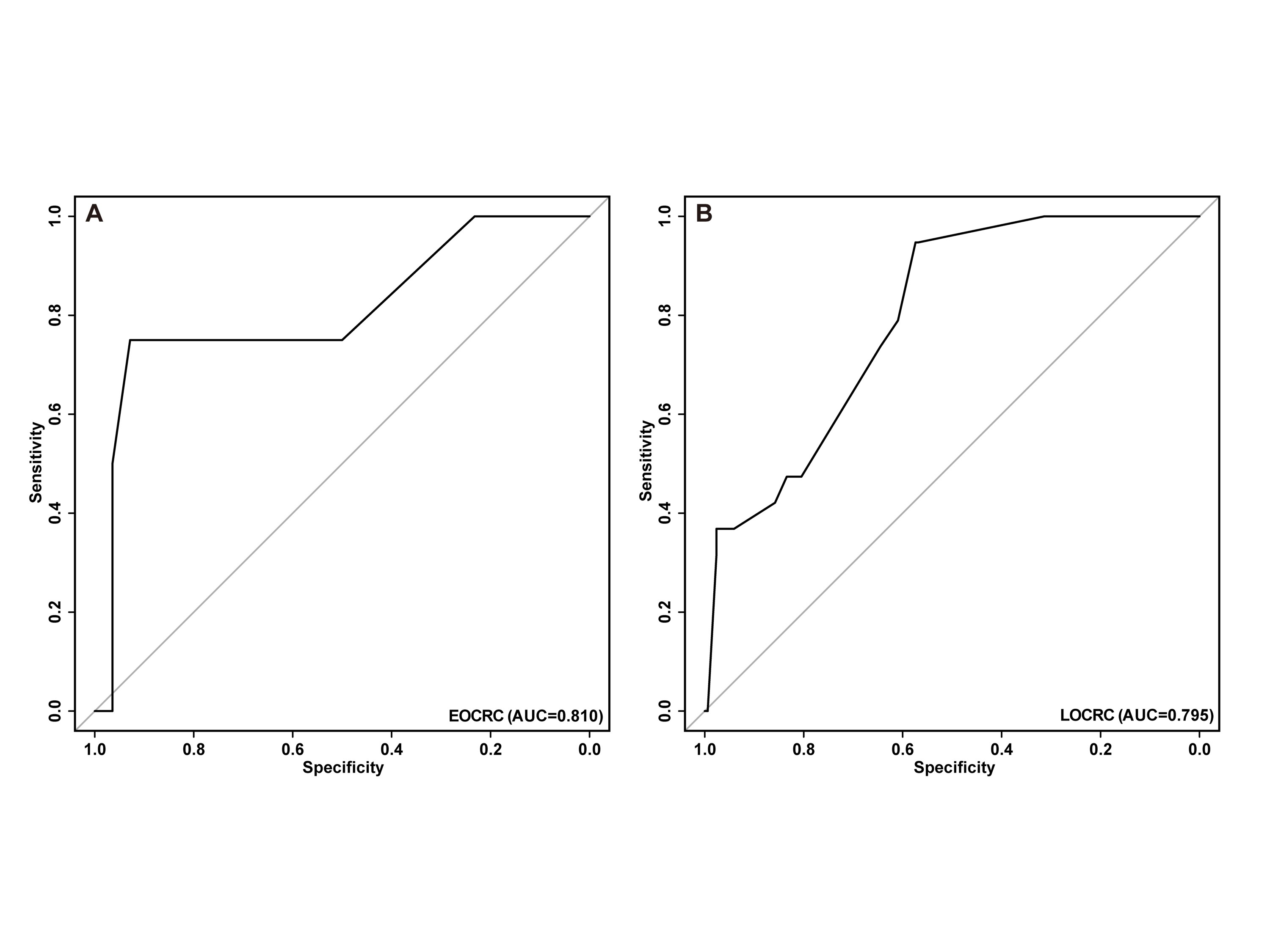

Supplement: Supplementary file 1 — Supplementary file1 (TIF 19796 KB) [file 384_2024_4621_MOESM1_ESM.tif]
